# Probiotic *Saccharomyces boulardii* Alleviates Lung Injury by Reduction of Oxidative Stress and Cytokine Response Induced by Supraceliac Aortic Ischemia-Reperfusion Injury in Rats

**DOI:** 10.21470/1678-9741-2020-0153

**Published:** 2021

**Authors:** Selim Durmaz, Tünay Kurtoğlu, Emin Barbarus, Nesibe Kahraman Çetin, Mustafa Yılmaz, Ömer Faruk Rahman, Filiz Abacıgil

**Affiliations:** 1 Department of Cardiovascular Surgery, Aydın Adnan Menderes University, Faculty of Medicine, Aydın, Turkey.; 2 Department of Medical Pathology, Aydın Adnan Menderes University, Faculty of Medicine, Aydın, Turkey.; 3 Department of Medical Biochemistry, Faculty of Medicine, Adnan Menderes University, Aydın, Turkey.; 4 Department of Public Health, Aydın Adnan Menderes University, Faculty of Medicine, Aydın, Turkey.

**Keywords:** Probiotics, Reperfusion Injury, Oxidative Stress, Cytokines, Lung Injury, Multiple Organ Failure, Saccharomyces boulardii

## Abstract

**Objectives:**

Ischemia-reperfusion injury is an important cause of multiple organ failure in cardiovascular surgery. Our aim is to investigate the effect of the probiotic *Saccharomyces boulardii* on oxidative stress, inflammatory response, and lung injury in an experimental model of aortic clamping.

**Methods:**

Twenty-one Wistar rats were randomized into three groups (n=7). Control group animals received saline gavage for a week before undergoing median laparotomy. In other groups, supraceliac aorta was clamped for 45 minutes to induce ischemia followed by reperfusion for 60 minutes. In the ischemia-reperfusion group, saline gavage was given preoperatively for one week. Ischemia-reperfusion+probiotic group rats received probiotic gavage for seven days before aortic clamping. The levels of oxidative stress markers and pro-inflammatory cytokines were determined in both serum and lung tissue samples. Ileum and lung tissues were harvested for histological examination.

**Results:**

Ischemia-reperfusion caused severe oxidative damage and inflammation evident by significant increases in malondialdehyde and cytokine levels (tumor necrosis factor alpha and interleukin-1 beta) and decreased glutathione levels in both serum and lung tissues. There was severe histological tissue damage to the lung and ileum in the ischemia-reperfusion group. Probiotic pretreatment before aortic clamping caused significant suppression of increases in serum and lung tissue malondialdehyde and tumor necrosis factor alpha levels. Histological damage scores in tissue samples decreased in the ischemia-reperfusion+probiotic group (*P*<0,005).

**Conclusions:**

Oral supplementation of probiotic *S. boulardii* before supraceliac aortic ischemia-reperfusion in rats alleviates lung injury by reducing oxidative stress, intestinal cellular damage, and modulation of inflammatory processes.

**Table:** 

Abbreviations, acronyms & symbols	
**ELISA**	**= Enzyme-linked immunosorbent assay**
**HPF**	**= High power fields**
**IL-1β**	**= Interleukin-1 beta**
**IR**	**= Ischemia-reperfusion**
**MDA**	**= Malondialdehyde**
**PBS**	**= Phosphate buffer**
**TNF-α**	**= Tumor necrosis factor alpha**

## INTRODUCTION

Ischemia-reperfusion injury is a serious consequence of open or endovascular treatment of abdominal aortic aneurysms, aortoiliac occlusive arterial diseases, aortic dissections, thoracic aortic aneurysms, and aortic trauma. Temporary supraceliac aortic clamping during surgery causes mesenteric ischemia. Restoration of the blood flow to affected tissues after the release of the clamp may trigger reperfusion injury mediated by the formation of free oxygen radicals, which paradoxically damages the cell membranes by lipid peroxidation^[^^1^^,^^2^^]^. Damage to the integrity of the intestinal mucosal barrier as a result of mesenteric ischemia may lead to translocation of enteric flora to mesenteric lymph nodes, liver, spleen and blood circulation^[^^3^^,^^4^^]^. As a result of these processes, a systemic inflammatory response is initiated, which can cause serious septic complications and consequently lead to multiple organ dysfunction and death^[^^5^^,^^6^^]^.

Probiotics are live microorganisms that provide health benefits to the host when given in reasonable amounts^[^^7^^]^. Microorganisms used as probiotics are a large family and include bacterial species such as *Lactobacillus, Bifidobacterium* and *Enterococcus* and fungi such as *Saccharomyces boulardii*. The beneficial effects of probiotics are associated with both regulation of the intestinal microbiota and direct interactions with host immunity^[^^8^^]^. Probiotics can restore the natural flora of the intestine and reinforce the resistance to colonization; therefore, it is suggested that they can be useful in pathological processes by suppressing microbial translocation and systemic inflammatory response^[^^9^^]^.

*S. boulardii* is non-pathogenic yeast with trophic and antimicrobial effects on the small intestine mucosa in humans and rats. *S. boulardii* has been shown to suppress the inflammatory responses experimentally, by decreasing the production of pro-inflammatory cytokines both *in vivo* and *in vitro* conditions^[^^10^^,^^11^^]^. It has also been demonstrated that dietary supplementation of *S. boulardii* reduces oxidative damage by supporting the antioxidant activity of tissues and preventing neutrophil accumulation^[^^12^^]^.

Therefore, the aim of our study is to investigate the effect of probiotic *S. boulardii* pretreatment on oxidative stress, inflammatory response and intestinal and lung injury in a supraceliac aortic occlusion model in rats.

## METHODS

### Animals and Groups

The animal care was conducted in accordance with the National Institute of Health's *Guide for the Care and Use of Laboratory Animals*. Adult female Wistar rats (5-6 months old) weighing 250-350 g were fed standard rat chow diet and water *ad libitum* and kept in cages in a temperature (22 °C±2 °C) and humidity (45-50%) controlled room with a 12-hour light-dark cycle and acclimatized for one week before the study. The experimental design and protocol were approved by the Animal Care Committee of Adnan Menderes University (approval number: 64583101/2019/032). The study was performed at Adnan Menderes University Faculty of Medicine, Experimental Animals Laboratory, Aydın, Turkey. A total of 21 animals were simply randomized into one of the following three groups, each consisting of seven animals: Control (C), Ischemia-Reperfusion (IR) and Ischemia-Reperfusion+Probiotic (IR+Pro) groups.

### Probiotic Pretreatment

*S. boulardii* is available in a lyophilized form (Reflor; Biocodex, Cedex, France) that contains 2.5×10^9^ live cells per sachet. The sachets were dissolved in saline and given at a dose of 500 mg/kg twice a day for seven days via gavage^[^^10^^,^^12^^]^. No adverse effects were observed in the probiotic-administered animals.

### Supraceliac Ischemia-Reperfusion Method

The rats received an intraperitoneal injection of ketamine 90 mg/kg (Ketalar; Parke Davis, Eczacibasi, Istanbul, Turkey) and xylazine 10 mg/kg (Rompun; Bayer AG, Leverkusen, Germany) mixture. After the induction of anesthesia, the abdomen was shaved and a median laparotomy was performed under sterile conditions. To maintain a body temperature of approximately 37 °C during the experiment, a heating lamp was used. Then, the abdominal aorta was explored and a non-traumatic vascular clamp (Vascu-Statt II, Scanlan, St. Paul, MN, USA) was placed in the supraceliac segment for 45 minutes, followed by reperfusion for 60 minutes as previously described^[^^1^^]^.

### Study Protocol

Control (C) Group (n=7): Saline gavage was applied for one week before the experimental surgery. The rats in this group underwent median laparotomy but aortic clamping and ischemia were not performed.

Ischemia-Reperfusion (IR) Group (n=7): Saline gavage was applied for one week before performing ischemia-reperfusion as described above.

Ischemia-Reperfusion+Probiotic (IR+Pro) Group (n=7): Probiotic pretreatment was given for one week before performing ischemia-reperfusion as described above.

### Blood and Tissue Sampling

At the end of the experiment, a median sternotomy was performed and 1 cc of blood was collected through right atrium puncture using a 30 G × 8 mm cannula, then the animals were sacrificed by exsanguination under deep anesthesia. Blood samples were centrifuged at 1,000 g for 10 minutes to obtain the serum and were stored at -80 °C until analysis. Segments of the ileum adjacent to the ileocecal valve were obtained for histological examination. The lung tissue was also harvested for biochemical and histological studies. Tissue samples harvested for histopathological examination were fixed in 10% phosphate-buffered formalin and the lung tissues that were sampled for biochemical studies were stored at -80 °C until analysis.

### Biochemical Analysis

Lung tissue samples were homogenized in 50 mM pH 7.4 phosphate-buffered saline (PBS) and were centrifuged at 20,000 g for 15 minutes. The supernatants were extracted and collected in a separate microcentrifuge tube for biochemical analysis. Tissue protein concentrations were measured by the method described by Lowry, based on the principle that the copper ion (Cu^+2^) forms a complex with peptide bonds in proteins and is reduced to Cu^+1^ in alkaline medium and the levels were detected spectrophotometrically at 660 nm (Shimadzu UV-160, CA, USA)^[^^13^^]^.

The concentrations of tumor necrosis factor alpha (TNF-α) and interleukin-1 beta (IL-1β) in serum and tissue samples were measured by performing enzyme-linked immunosorbent assay (ELISA) analyses. Inflammatory mediators from each sample were quantified using rat specific ELISA kits (E-EL-R0019-E-EL-R0012, Elabscience Biotechnology Co., Wuhan, PRC). Results were calculated using ELISA microplate reader (DAR 800, Diagnostic Automation, CA, USA) using standard curves and expressed as picograms per milliliter (pg/mL) of serum.

The malondialdehyde (MDA) levels were determined using the method previously described by Ohkawa et al.^[^^14^^]^; the results were expressed as µmol/mg of protein for tissue samples and nmol/ml for serum samples. The glutathione levels were detected using the method described by Beutler et al.^[^^15^^]^; the results were expressed as µmol/mg protein for tissue samples and µmol/L for serum samples.

### Histopathologic Evaluation

The ileal and lung tissue samples were embedded in paraffin blocks and 4-µm thick sections were obtained and stained with hematoxylin-eosin. The sections were examined under light microscope (Olympus BX53, Olympus Co., Tokyo, Japan) by a blinded pathologist.

The histopathological damage score for ileal tissue samples was calculated based on the villus height, frequency of inflammation in the *lamina propria*, changes in mucosal architecture (general structure, cellular distribution, mucosal and submucosal aspect), and the presence of ulcerations. The following scale was used for each parameter: absent (0), mild (1), moderate (2), and intense (3). The results are presented as the sum of the scores obtained for each parameter^[^^16^^]^.

The lung tissue samples were evaluated based on the alveolar wall thickening and intraalveolar edema. The alveolar wall thickening was graded as "0" when less than two cell layers thick or "1" when greater. Intraalveolar edema, defined as homogenous or fibrillary proteinaceous staining within the alveoli, was graded as "0" when absent or "1" when present.

The number of neutrophils was noted in each of the randomly sampled five high-power fields (HPF) (× 400 magnifications). The total number of neutrophils was counted in each of the five HPFs and expressed as the total number/5HPF for each rat^[^^17^^]^.

### Statistical Analysis

The measurements were analyzed using the SPSS software (v. 24.0, IBM Corp., Armonk, NY, USA). The descriptive statistics of the groups were calculated as frequency and percentage values to examine the relevant data. The Shapiro-Wilk test was used to evaluate whether the distribution of continuous variables was normal. As the continuous variables were normally distributed, descriptive analyses were presented as mean±standard deviation. One-way ANOVA was used to compare parameters among study groups. Levene's test was used to assess the homogeneity of the variances. Tukey's post-hoc test was performed to determine the significance of pairwise differences using the Bonferroni correction to adjust for multiple comparisons. A 5% type 1 error level was used to infer statistical significance.

## RESULTS

### Serum and Tissue Oxidative Stress Parameters

**Fig. 1:**
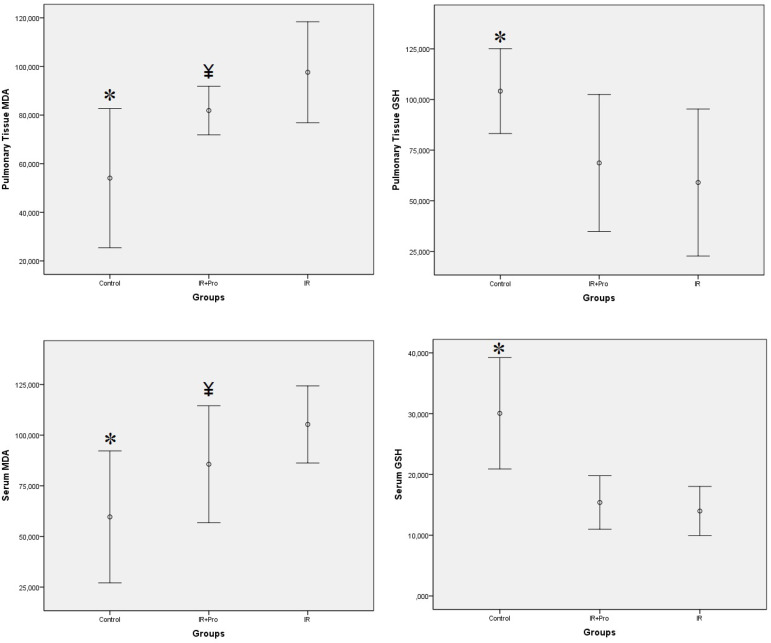
Results of serum and lung tissue oxidative stress parameters. *P<0.05 compared to the IR and IR+Pro groups. ^¥^P<0.05 compared to the IR group. IR=ischemia-reperfusion; IR+Pro=ischemia-reperfusion+probiotic. GSH=glutathione; MDA=malondialdehyde

Levels of serum and lung tissue oxidative stress parameters among the study groups are demonstrated in Figure 1. The serum and tissue glutathione levels were significantly lower in the IR and IR+Pro groups in comparison to the Control group (*P*<0.05).

Serum and tissue malondialdehyde levels were significantly lower in the control group in comparison to the other two groups (*P*<0.05).

There were no significant differences regarding the GSH levels among IR and IR+Pro groups (*P*>0.05). On the other hand, both serum and tissue MDA levels were significantly lower in the IR+Pro group as compared to the IR group (*P*<0.05).

### Serum and Tissue Cytokine Levels

**Fig. 2:**
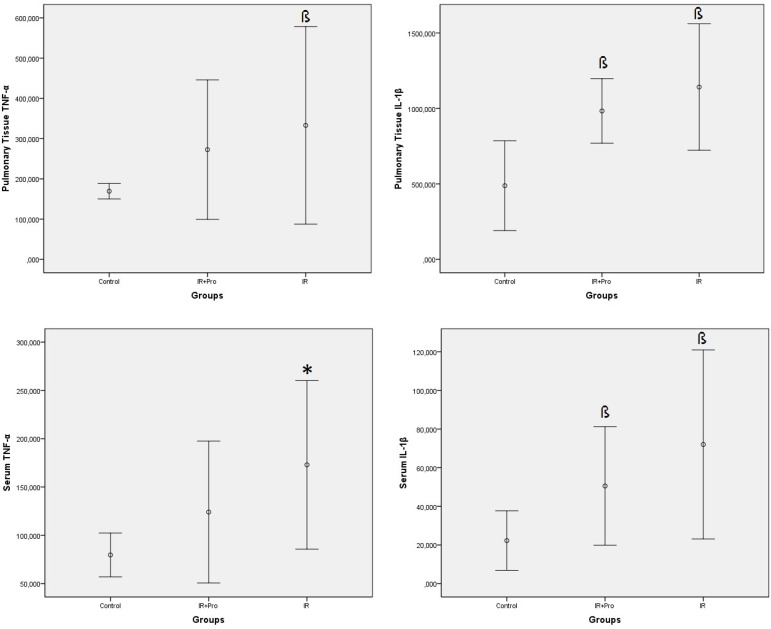
Results of serum and lung tissue cytokine levels. *P<0.05 compared to the control and IR+Pro groups. ^ß^P<0.05 compared to the control group. IR=ischemia-reperfusion; IR+Pro=ischemia-reperfusion+probiotic. TNF-α=tumor necrosis factor alpha; IL-1β=interleukin 1 beta

Serum and lung tissue cytokine levels among study groups are demonstrated in Figure 2. Serum TNF-α levels were significantly increased in the IR group compared to the other study groups (*P*<0.05). However, there were no significant differences regarding the serum TNF-α levels between the control and IR+Pro groups (*P*>0.05). TNF-α levels in lung tissue increased significantly in the IR group compared to the control group (*P*<0.05). On the other hand, the tissue TNF-α levels in the IR+Pro group were not significantly increased in comparison to the control group (*P*>0.05).

The serum and tissue IL-1β levels were significantly higher in the IR and IR+Pro groups compared to the control group (*P*<0.05). Although there were no significant differences regarding the tissue IL-1β levels among IR and IR+Pro groups, the levels were higher in the IR group (*P*>0.05).

### Histologic Evaluation

**Fig. 3:**
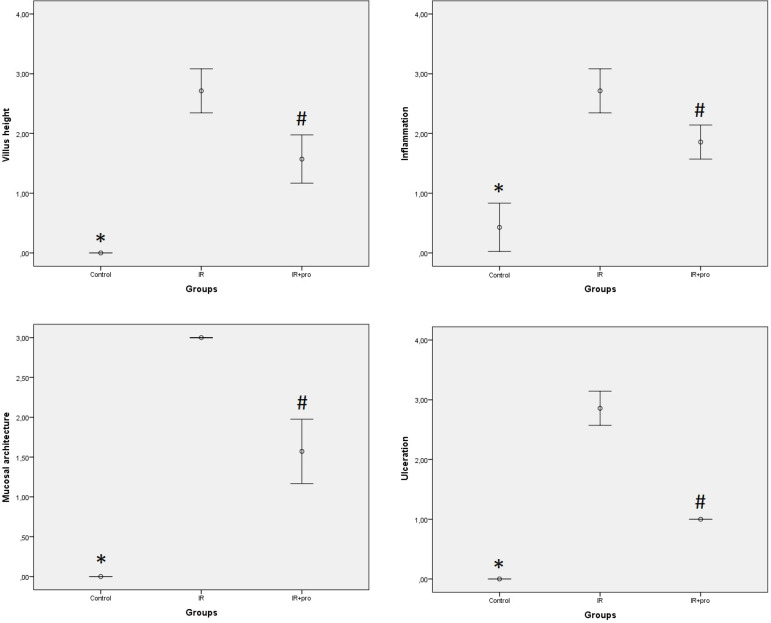
Results of histopathological damage scores of ileal tissue. *P<0.05 compared to the IR and IR+Pro groups. ^#^P<0.05 compared to the IR group.

**Fig. 4:**
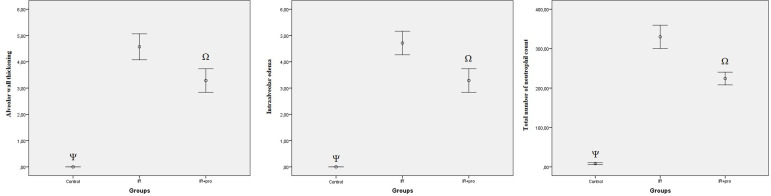
Results of histopathological grades of lung tissue and the total number of neutrophil counts. ^Ψ^P<0.05 compared to the IR and IR+Pro groups. ^Ω^P<0.05 compared to the IR group.

The results of histopathological damage scores of ileal tissue samples are shown in Figure 3. Histopathological damage scores in the IR and IR+Pro groups were significantly higher than the scores in the Control group (*P*<0,05). In the IR+Pro group, histopathological damage scores decreased significantly compared to the scores of IR group (*P*<0.05). Histopathological grades of lung tissue samples and the total number of neutrophil counts are shown in Figure 4. The total number of neutrophil counts and the histopathological grades of intraalveolar edema and alveolar wall thickening were significantly higher in IR and IR+Pro groups in comparison to the control group (*P*<0.05). In the IR+Pro group, histopathological grades and the total number of neutrophil counts decreased significantly as compared to the IR group (*P*<0.05).

**Fig. 5:**
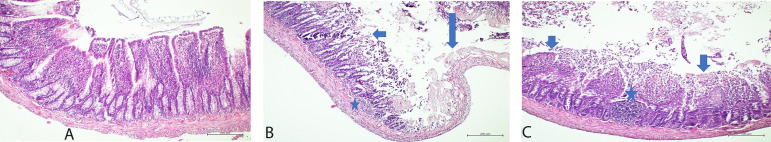
Representative histological samples of ileal tissue. Photomicrographs of ileal tissue samples (100x magnification, H-E staining). A) Control group. B) IR group. C) Probiotic group. The long arrow points to areas of ulceration and tissue damage, the short arrow points to reduction of villus height and the points asterisk to inflammatory cells.

**Fig. 6:**
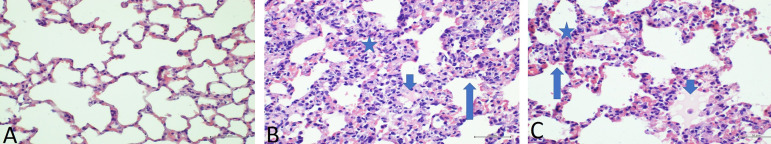
Representative histological samples of lung tissue. Photomicrographs of lung tissue samples (400x magnification, H-E staining). A) Control group. B) IR group. C) Probiotic group. The long arrow points to areas of thickened and congested alveolar walls, the short arrow points to intraalveolar fluid and the asterisk points to inflammatory cells.

Representative histological sections of ileal and lung tissue from study groups are sequentially shown in Figures 5 and 6. Ileal tissue samples in the control group presented the preservation of the villi and epithelium (Figure 5A). Histopathological examination of the IR group revealed tissue damage characterized by mucositis, a reduction in villus height, presence of inflammatory cells and ulcerations (Figure 5B). On the other hand, the IR+Pro group demonstrated preservation of villi length, lack of intense mucosa ulcerations and overall reduced tissue damage (Figure 5C). The lung tissue samples from the control group displayed typical histology of normal lungs with thin alveolar walls and absence of intraalveolar edema (Figure 6A). In the IR group, thickened and congested alveolar walls, intraalveolar edema, and marked leukocyte infiltration, consistent with acute lung injury, were observed (Figure 6B). However, in the IR+Pro group, fewer thickened alveolar walls, less intraalveolar edema and fluid, and fewer neutrophils were detected (Figure 6C).

## DISCUSSION

In our study, we used supraceliac aortic clamping to induce ischemia-reperfusion injury in rats. Ischemia-reperfusion caused an increase in malondialdehyde levels and a decrease in glutathione levels in both serum and lung tissues, which indicates severe oxidative damage. We also found an increase in cytokine (TNF-α and IL-1β) levels in serum and lung tissue, which indicates that a prominent inflammatory response is triggered as a result of aortic clamping. Additionally, ischemia-reperfusion resulted in severe histological damage to the intestine and lung tissues in our experimental model. Oral supplementation of *S. boulardii* before aortic clamping caused suppression of increases in serum and lung tissue malondialdehyde and TNF-α levels, which are accompanied by considerable attenuation of the severity of histological damage in the small intestine and lung tissues. Our findings suggest that *S. boulardii* pretreatment in experimental ischemia-reperfusion exerts beneficial effects by alleviating oxidative stress and cytokine release responses in circulating blood and lungs. The alleviation of the lung damage in animals which were given probiotic supplementation may be associated with the following: 1) reduction of oxidative stress; 2) reduction of intestinal cellular damage; 3) limitation of the pro-inflammatory cytokine response.

Acute lung injury is one of the most important causes of morbidity and mortality following cardiovascular procedures, especially those involving clamping of the thoracic and/or abdominal aorta^[^^18^^]^. The main mechanisms of organ failure induced by aortic clamping-including the lungs-are the provocation of systemic oxidative stress and inflammatory response that are related to tissue hypoxia^[^^1^^,^^19^^]^. The local ischemic effects on the intestinal mucosa play an essential role in this process and in the resulting lung injury. Inadequate mucosal blood flow during aortic occlusion causes direct damage to the intestinal cells, impairing gut barrier functions, thereby leading to the invasion of bacteria or endogenous endotoxins^[^^20^^]^. Disruption of the integrity of intestinal epithelial cells enhances the activation of systemic inflammatory mediators, including TNF-α and interleukins. These pro-inflammatory cytokines produced by the immune cells as part of the overall host reaction play a leading role in the lung injury that follows intestinal ischemia-reperfusion^[^^21^^,^^22^^]^. The pulmonary tissue injury is typically apparent as alveolar cell damage, inflammatory cell infiltration, and neutrophil accumulation^[^^19^^]^.

In this study, we found that reperfusion following supraceliac aortic clamping in rats resulted in severe histological damage in lungs, which is associated with systemic oxidative stress, pro-inflammatory cytokine release, and loss of the cellular integrity in the intestinal tissue. Similar findings have also been reported previously in various experimental studies of supraceliac aortic ischemia-reperfusion in literature^[^^1^^,^^19^^,^^21^^]^.

Probiotics can support the natural physical barrier functions by helping to protect the integrity of intestinal epithelial cells. *S. boulardii* is a probiotic commonly employed in the treatment of infectious diarrhea and is well known for supporting intestinal mucosal barrier in such conditions^[^^9^^]^. Moreover, this probiotic is also thought to offer potential cytoprotective effects in other pathologic conditions that can threaten the intestinal mucosa^[^^23^^]^. Preoperative administration of *S. boulardii* has been shown to preserve villus height, maintain mucosal integrity and reduce bacterial translocation in an experimental model of intestinal obstruction^[^^24^^]^. *S. boulardii* treatment has also been demonstrated to reduce the epithelium and villus degeneration and block the infiltration of inflammatory cells in the ileum during drug-induced oxidative damage in rats^[^^12^^]^. In the present study, we have observed that *S. boulardii* pretreatment protected small intestine against aortic ischemia-reperfusion injury by conserving the villus height and mucosal architecture and decreasing inflammation.

Although the exact mechanisms are not fully understood, *S. boulardii*, used in our study, has many effects on the local and systemic immune systems. It has been shown to modulate inflammatory responses through suppression of pro-inflammatory mediators, via secreted factors interfering with signaling pathways or by acting directly as an immune stimulant^[^^9^^,^^23^^]^. In an *in vitro* study, the addition of *S. boulardii* to intraepithelial lymphocyte cultures stimulated by microbial pathogens reduced the release of pro-inflammatory cytokines^[^^11^^]^. Karen et al.^[^^10^^]^ have demonstrated that *S. boulardii* decreases the cytokine release and attenuates the histologic signs of lung injury in an experimental acute pancreatitis model. In our study, *S. boulardii* supplementation caused a significant suppression of TNF-α levels in serum and lung tissue in aortic ischemia-reperfusion. Although the IL-1β levels in serum and lung tissues were also decreased due to probiotic pretreatment, the changes did not reach statistical significance. These results suggest that *S. boulardii* affected the inflammatory response by limiting the release of pro-inflammatory cytokines.

There is also evidence that *S. boulardii* can support antioxidant defenses in the course of oxidative tissue injury. Akyol et al.^[^^25^^]^ demonstrated that *S. boulardii* supplementation reduces the erythrocyte malondialdehyde levels and they also observed that, when used in conjunction with antibiotics, it significantly prevents oxidative stress in an acute pancreatitis model. Furthermore, *S. boulardii* has been shown to ameliorate oxidative intestinal damage and hepatic inflammation by increasing the antioxidant state of the tissues and inhibiting neutrophil recruitment to the affected tissues^[^^9^^]^. In the present study, we observed that increases in serum and lung tissue malondialdehyde levels related to aortic ischemia-reperfusion were significantly suppressed by *S. boulardii* supplementation. However, the serum and tissue glutathione levels were not significantly affected by the probiotic pretreatment. The reason that glutathione levels are not significantly changed might be the severity of the ischemia-reperfusion induced in our animal model. Although these results suggest that *S. boulardii* decreases oxidative stress to some extent, the exact mechanisms are not clear.

Certain limitations should be considered while interpreting the results of our study: first, although we have examined the structural integrity of intestinal epithelium by histology, we did not evaluate the physiologic functions by gut permeability tests or the amount of bacterial translocation to adjacent tissues. Second, the determination of oxidative stress markers and cytokines in the intestine could have yielded additive information regarding the mechanism of action of the probiotic supplementation.

## CONCLUSION

In brief, the results of this study indicated that *S. boulardii* supplementation prior to supreceliac aortic clamping alleviates ischemia-reperfusion-induced lung injury in rats. We think that this beneficial effect might be related to the attenuation of intestinal cellular damage, reduction of cytokine response, and the suppression of oxidative stress. However, further investigation is needed to determine the exact effects of *S. boulardii* supplementation in acute ischemic conditions.

**Table:** 

Authors' roles & responsibilities	
SD	Substantial contributions to the conception or design of the work; or the acquisition, analysis or interpretation of data for the work; drafting the work or revising it critically for important intellectual content; final approval of the version to be published
TK	Substantial contributions to the conception or design of the work; or the acquisition, analysis or interpretation of data for the work; drafting the work or revising it critically for important intellectual content; final approval of the version to be published
EB	Substantial contributions to the conception or design of the work; or the acquisition, analysis or interpretation of data for the work; drafting the work or revising it critically for important intellectual content; final approval of the version to be published
NKÇ	Substantial contributions to the conception or design of the work; or the acquisition, analysis or interpretation of data for the work; drafting the work or revising it critically for important intellectual content; final approval of the version to be published
MY	Substantial contributions to the conception or design of the work; or the acquisition, analysis or interpretation of data for the work; drafting the work or revising it critically for important intellectual; final approval of the version to be published
ÖFR	Substantial contributions to the conception or design of the work; or the acquisition, analysis or interpretation of data for the work; drafting the work or revising it critically for important intellectual content; final approval of the version to be published
FA	Substantial contributions to the conception or design of the work; or the acquisition, analysis or interpretation of data for the work; drafting the work or revising it critically for important intellectual content; final approval of the version to be published
